# Kiwifruit genomics and applications: recent advances, current challenges, and future prospects

**DOI:** 10.1093/hr/uhag024

**Published:** 2026-01-29

**Authors:** Malik Umair Faiz, Xin Liu, Jiarui Sun, Cecilia H Deng, Yanfei Liu, Xinxin Wang, Zihan Fan, Xueying Hong, Lihuan Wang, Wei Li, Wei Tang, Pu Liu, Yang Song, Xiujuan Qi, Dawei Li, Xueren Yin, Yongsheng Liu, Junyang Yue

**Affiliations:** Anhui Province Key Laboratory of Horticultural Crop Quality Biology, School of Horticulture, Anhui Agricultural University, Hefei 230036, China; Anhui Province Key Laboratory of Horticultural Crop Quality Biology, School of Horticulture, Anhui Agricultural University, Hefei 230036, China; Anhui Province Key Laboratory of Horticultural Crop Quality Biology, School of Horticulture, Anhui Agricultural University, Hefei 230036, China; Mt Albert Research Centre, New Zealand Institute of Bioeconomy Science Limited, Auckland 1142, New Zealand; College of Horticulture, Northwest A&F University, Yangling 712100, China; Anhui Province Key Laboratory of Horticultural Crop Quality Biology, School of Horticulture, Anhui Agricultural University, Hefei 230036, China; Anhui Province Key Laboratory of Horticultural Crop Quality Biology, School of Horticulture, Anhui Agricultural University, Hefei 230036, China; Anhui Province Key Laboratory of Horticultural Crop Quality Biology, School of Horticulture, Anhui Agricultural University, Hefei 230036, China; Anhui Province Key Laboratory of Horticultural Crop Quality Biology, School of Horticulture, Anhui Agricultural University, Hefei 230036, China; Anhui Province Key Laboratory of Horticultural Crop Quality Biology, School of Horticulture, Anhui Agricultural University, Hefei 230036, China; Anhui Province Key Laboratory of Horticultural Crop Quality Biology, School of Horticulture, Anhui Agricultural University, Hefei 230036, China; Anhui Province Key Laboratory of Horticultural Crop Quality Biology, School of Horticulture, Anhui Agricultural University, Hefei 230036, China; Research Institute of Pomology, Chinese Academy of Agricultural Sciences/Key Laboratory of Horticultural Crops Germplasm Resources Utilization, Ministry of Agriculture and Rural Affairs, Xingcheng 125100, China; National Key Laboratory for Germplasm Innovation & Utilization of Horticultural Crops, Zhengzhou Fruit Research Institute, Chinese Academy of Agricultural Sciences, Zhengzhou 450009, China; Key Laboratory of Plant Germplasm Enhancement and Specialty Agriculture, Wuhan Botanical Garden, The Chinese Academy of Sciences, Wuhan 430074, China; Anhui Province Key Laboratory of Horticultural Crop Quality Biology, School of Horticulture, Anhui Agricultural University, Hefei 230036, China; Anhui Province Key Laboratory of Horticultural Crop Quality Biology, School of Horticulture, Anhui Agricultural University, Hefei 230036, China; Anhui Province Key Laboratory of Horticultural Crop Quality Biology, School of Horticulture, Anhui Agricultural University, Hefei 230036, China; Research Center for Biological Breeding, Advanced Academy, Anhui Agricultural University, Hefei 230036, China

## Abstract

Kiwifruit (*Actinidia* spp.) is a globally significant horticultural crop, renowned for its exceptional nutritional value and high vitamin C content. The distinctive genetic features of this genus, including a dioecious sexual system (XY/XX) and a wide range of ploidy (2*x*–10*x*), have driven substantial genomic and phenotypic diversification, thereby constituting a valuable germplasm resource for systematic breeding. Recent advances in kiwifruit genomics are transforming the field and revolutionizing our understanding of its evolution, domestication, and the genetic mechanisms underlying agronomic traits. In this review, we highlight the key achievements in kiwifruit genome research over the past decades, chronologically spanning from the initial draft genome assembly to the recent super pan–genome construction. We further synthesize how multi-omics approaches have been leveraged for fine mapping, gene discovery, and the analysis of gene expression and metabolic pathways. Finally, we discuss future research directions and breeding strategies enabled by these genomic breakthroughs, particularly through the applications of genomic selection and gene editing in kiwifruit.

## Introduction

Kiwifruit, botanically classified under the genus *Actinidia* in the family Actinidiaceae, is widely distributed throughout most regions of East Asia [1]. The center of origin is thought to be the Yangtze River valley and adjacent mountain ranges in central and eastern China. As of now, 54 kiwifruit species have been formally documented [2], though taxonomic revisions may lead to updates in the future. Notably, these confirmed species exhibit striking ploidy variation ranging from diploids (2*x*) to tetraploids (4*x*), hexaploids (6*x*), octoploids (8*x*), and even decaploids (10*x*), which profoundly enhances both genomic and phenotypic diversities within the genus [3, 4]. Moreover, all kiwifruit species are functionally dioecious with separate male and female vines. This reproductive system inherently facilitates both spontaneous hybridization and adaptive introgression in natural populations, leading to significantly greater interspecific and intraspecific genetic variation in kiwifruit relative to monoecious or self-compatible crops [5].

Despite the availability of rich kiwifruit germplasm resources, only a few species, such as *Actinidia chinensis*, *Actinidia deliciosa*, *Actinidia arguta,* and *Actinidia eriantha*, have been selectively domesticated and commercially cultivated to date [6]. The limited number of economically viable kiwifruit species can be attributed to a relatively short domestication history, with systematic commercial cultivation only beginning after its transfer from China to New Zealand in 1904 [7]. Nevertheless, these cultivated species have undergone remarkably rapid global expansion, transitioning from wild Chinese vines to internationally traded commodities within just a century. Today, kiwifruit ranks among the world’s most popular fresh fruits, with annual production exceeding four million tons across more than 30 countries (https://www.fao.org/faostat/en/#home, 2023). Furthermore, the international market still continues to expand, primarily driven by the iterative improvement of novel kiwifruit cultivars. Technically speaking, this swift commercial success is fundamentally attributable to their outstanding nutritional profiles and distinctive flavor characteristics [8]. Especially, owing to the exceptionally high vitamin C content, kiwifruit is commonly celebrated as ‘the king of vitamin C’ and ‘the king of fruits’. In addition to fresh consumption, kiwifruit also delivers medicinal benefits (particularly in root tissues) and ornamental uses (notably in flowering vines), demonstrating untapped potential for value-added utilization [9].

Over the past decades, ongoing research in kiwifruit encompasses multidisciplinary investigations, spanning sex determination, juvenile stage, plant architecture, flowering time, fruit characteristics, biochemical composition, environmental adaptation, and disease tolerance [10, 11]. Concurrently, the continuing progression of sequencing technologies have accelerated the generation of multi-omics data encompassing genomic (DNA), transcriptomic (RNA), proteomic (protein), metabolomic (metabolite), and epigenomic (epigenetic) profiles across wild species and cultivated varieties, which significantly advances our understanding of the genetic basis of phenotypic traits. In particular, the publication of the first reference genome in 2013 marked a pivotal breakthrough in kiwifruit genomics and established an essential framework for systematic investigations into comparative genomic analysis, genetic diversity characterization, gene family evolution, gene expression profiling, protein composition analysis, complex biochemical changes, biological pathway construction, and molecular marker development at the system level [12]. Furthermore, the availability of bioinformatics tools and data resources has offered new opportunities for the resequencing of cultivated and wild kiwifruit accessions, enabling the discovery of trait-associated variants that facilitate gene editing-assisted breeding programs [13, 14].

In this review, we systematically summarize the major breakthroughs in multi-dimensional omics, including genomic, epigenomic, transcriptomic, proteomic, metabolomic studies that have reshaped our mechanistic understanding of kiwifruit biology. Recent advancements in genomic resources not only offer new insights into the genetic diversity and evolutionary dynamics of the genus *Actinidia* but also enable precise resolution of the spatiotemporal genotype–phenotype relationships for various economic and desirable agronomic traits in kiwifruit, which paves the way for their targeted engineering via cutting-edge genome editing technologies. Moreover, we discuss the current challenges and future perspectives in kiwifruit genomics research, particularly highlighting potential strategies to accelerate breeding programs.

## Omics advances: from high-throughput methodologies to biological insights

### Genome sequencing and assembly

**Figure 1:**
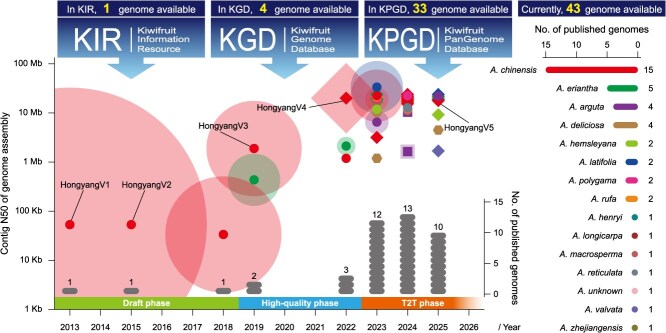
Schematic of the entire process for kiwifruit genome sequencing and assembly, with three phases defined by improvements in genome integrity and contiguity. Colors represent different species, as indicated in the right sidebar. Shapes denote the level of haplotype resolution: circle (one haplotype), diamond (two haplotypes), square (four haplotypes), and hexagon (six haplotypes). The radiation area corresponds to citation counts (as of 28 August 2025). The number of genomes published per year and per species is summarized. Notably, three major genome databases, including KIR, KGD, and KPGD, are highlighted. Furthermore, all versions of the ‘Hongyang’ genome assembly are annotated.

The genome sequencing has been a pivotal step in elucidating the roles of genetic factors that contribute to the diversity of phenotypic traits. In 2011, a feasible plan to decode the complete genome sequence of kiwifruit has been brought forward by the International Kiwifruit Genome Consortium (IKGC), which was composed by scientists from Hefei University of Technology, Sichuan University, Cornell University, and Anhui Agricultural University, as well (https://kir.atcgn.com/news.html). After a comprehensive assessment, one female individual of the elite cultivar ‘Hongyang’ from *A. chinensis* (2*n* = 2*x* = 58) was selected for kiwifruit whole-genome sequencing [12]. Particularly, sequencing was run on the Illumina HiSeq 2000 system and accomplished in early 2012 with the acquisition of 105.8 Gb paired-end sequences corresponding to ~140× genome coverage. As expected, the following *de novo* assembly yielded a draft genome of 616.1 Mb, with the contig N50 size of 58.8 Kb (Fig. 1 and Table 1). Based on a high-density genetic map, a total of 452.4 Mb sequences, accounting for ~73.4% of the assembled genome, were successfully anchored to all of the 29 pseudochromosomes. Finally, the genome assembly (hereafter HongyangV1) along with its structural and functional annotations was released on September 19, 2013, making kiwifruit the first sequenced species in the Actinidiaceae family, even in the Ericales order [12]. Importantly, two recent whole-genome duplication (WGD) events after the divergence of kiwifruit and Solanaceae lineages were identified through comparative genomic analysis, which may contribute to the neofunctionalization of duplicated genes underlying high vitamin C accumulation in the fruit of kiwifruit.

**Table 1:** Summary statistics of genomic features for the currently published kiwifruit genomes

**Pub Date**	**Genome ID** ^**a**^	**Material name**	**Species name** ^**a**^	**Gender**	**Ploidy**	**Phasing**	**Anchored size**	**Contig N50**	**Gene number** ^**b**^	**Reference**
2013-10-18	HongyangV1	Hongyang	*A. chinensis*	F	2x	1	454.1 Mb	58.8 Kb	39 040	[12]
2015-11-05	HongyangV2	Hongyang	*A. chinensis*	F	2x	1	454.1 Mb	58.8 Kb	39 761	[15]
2018-04-10	Red5V1	Red5	*A. chinensis*	F	2x	1	548 Mb	33.8 Kb	33 044	[16]
2019-03-01	WhiteV1	White	*A. eriantha*	F	2x	1	682.7 Mb	539.2 Kb	42 988	[17]
2019-09-02	HongyangV3	Hongyang	*A. chinensis*	F	2x	1	583.2 Mb	1.7 Mb	40 464	[18]
2022-05-08	MaohuaW1	wild	*A. eriantha*	F	2x	1	629.8 Mb	2.0 Mb	41 521	[19]
2022-05-16	RussellV1	Russell	*A. chinensis*	M	2x	1	618.6 Mb	1.3 Mb	33 833	[20]
2022-11-21	HongyangV4	Hongyang	*A. chinensis*	F	2x	2	606.1/599.6 Mb	19.0/18.0 Mb	45 809/45434	[21]
2023-01-30	MidaoV1	Midao31	*A. eriantha*	F	2x	2	619.4/611.7 Mb	21.0/18.0 Mb	46 008/47184	[22]
2023-01-31	GezaoW1	wild	*A. polygama*	M	2x	1	618.2 Mb	19.0 Mb	44525^K^	[23]
2023-01-31	RuanzaoW1	wild	*A. arguta*	M	2x	1	636.5 Mb	7.1 Mb	48087^K^	[23]
2023-01-31	ShanliW1	wild	*A. rufa*	M	2x	1	620.3 Mb	20.1 Mb	46070^K^	[23]
2023-02-06	DonghongV1	Donghong	*A. chinensis*	F	2x	1	608.3 Mb	20.6 Mb	42 685	[24]
2023-02-06	KuoyeW1	wild	*A. latifolia*	F	2x	1	640.6 Mb	22.5 Mb	41 317	[24]
2023-02-07	MeiweiW1	wild	*A. deliciosa*	F	2x	1	619.9 Mb	10.1 Mb	38 990	[25]
2023-05-31	ChangyeW1	wild	*A. hemsleyana*	F	2x	1	637.7 Mb	13.3 Mb	42 538	[26]
2023-05-31	ZhejiangW1	wild	*A. zhejiangensis*	F	2x	2	615.1/630 Mb	18.2/18.2 Mb	45 869/46486^K^	[26]
2023-09-19	MeiweiW2	wild	*A. deliciosa*	F	6x	6	538.1/583.3/593.3/591.4 /571.5/595.7 Mb	1.3/1.6/1.7/1.6 /1.4/1.6 Mb	34 896/36324/37175 /37675/36740/37498^K^	[27]
2023-10-31	BlankV1	Blank	*A. eriantha*	M	2x	2	619.4/613.6 Mb	18.4/11.3 Mb	45 135/46105	[28]
2023-10-31	H0809V1	H0809	*A. chinensis*	M	2x	2	605/600.3 Mb	3.2/2.8 Mb	46 252/45170	[28]
2024-02-06	Longcheng2V1	Longcheng2	*A. arguta*	F	4x	4	615.2/595/570.3 /552.7 Mb	1.5/1.3/1.2 /1.0 Mb	40 859/41377/39833 /39222	[29]
2024-05-17	BiyuV1	Biyu	*A. chinensis*	F	2x	2	602/597.9 Mb	20.3/19.0 Mb	46 073/46634	[30]
2024-05-17	Hort16AV1	Hort16A	*A. chinensis*	F	2x	2	602.7/601.3 Mb	19.2/19.4 Mb	44 336/46230	[30]
2024-05-17	HuangyangV1	Huangyang	*A. chinensis*	F	2x	2	604.7/601 Mb	19.1/17.6 Mb	45 982/45004	[30]
2024-05-17	JinmiV1	Jinmi	*A. chinensis*	F	2x	2	604.5/595.6 Mb	20.4/18.6 Mb	45 473/45101	[30]
2024-05-17	JinpaiV1	Jinpai	*A. chinensis*	F	2x	2	603.3/592.7 Mb	18.1/15.6 Mb	46 244/45675	[30]
2024-05-17	Zps18V1	Zps18	*A. chinensis*	F	2x	2	602.7/603.1 Mb	18.2/20.1 Mb	45 873/45481	[30]
2024-06-10	RuanzaoW2	wild	*A. arguta*	M	4x	4	658.8/652.8/654.6 /649.4 Mb	10.9/9.6/10.3 /12 Mb	42 001/42216/41759 /41585^K^	[31]
2024-09-11	ChangguoW1	wild	*A. longicarpa*	F	2x	1	631.6 Mb	15.5 Mb	47014^K^	[32]
2024-09-11	DaziW1	wild	*A. macrosperma*	F	4x	1	576.4 Mb	11.2 Mb	43756^K^	[32]
2024-09-11	GezaoW2	wild	*A. polygama*	F	2x	1	612 Mb	19.2 Mb	43213^K^	[32]
2024-09-11	ShanliW2	wild	*A. rufa*	F	2x	1	613.7 Mb	12.2 Mb	47011^K^	[32]
2024-09-11	WangmaiW1	wild	*A. reticulata*	F	2x	1	624.2 Mb	14.7 Mb	45478^K^	[32]
2025-03-03	ChangyeW2	wild	*A. hemsleyana*	F	2x	2	634.8/634.8 Mb	8.4/9.0 Mb	41 466/41649	[33]
2025-03-03	HongyangV5	wild	*A. chinensis*	F	2x	2	610.4/602.0 Mb	19.5/18.0 Mb	41 550/41368	[33]

(Continued)

To facilitate the sharing of whole genome data, IKGC has constructed a comprehensive database for kiwifruit genomics (Kiwifruit Information Resource [KIR]) immediately [15]. It should be noted that the protein-coding genes were re-predicted by integrating transcript profiles and splice variants available across multiple transcriptome studies, leading to the change of both gene numbers and gene models. More precisely, 21 132 existing and 9547 novel transcripts have been modified and detected, respectively, which yielded a total of 39 761 predicted genes in the assembled genome (Table 1). To distinguish it from HongyangV1, the kiwifruit genome data in KIR was named HongyangV2 in order (Fig. 1). Currently, the website address of KIR has changed to https://kir.atcgn.com/ with no disruption to the ongoing service.

As the first sequencing material for kiwifruit genome and one of the most widely planted cultivar in China, *A. chinensis* ‘Hongyang’ has been a major focus of attention in kiwifruit researches. This can also be seen from its iterative updates of genome sequencing and assembly, which are always continuously aligning with the development of advanced sequencing technologies. Based on Pacific Biosciences (PacBio) long reads and Hi-C reads, an improved chromosome-level genome assembly was released in 2019 and named HongyangV3 [18]. HongyangV3 has an obvious improvement in integrity, continuity, and accuracy over the two previous versions. Specially, the use of new sequencing technology has enabled the volume increment of contig N50 value from 58.8 Kb to 1.72 Mb when assembling the HongyangV3 genome (Fig. 1 and Table 1). Meanwhile, the high-quality of HongyangV3 allows the analysis of long terminal repeat (LTR) retrotransposons across the genome, revealing 1212 intact LTR-RTs with insertion times within one million years.

With the rapid progress of PacBio HiFi and Oxford ONT ultra-long technologies, genome assembly has quickly entered the era of ‘telomere-to-telomere’ (T2T). In 2022, the genome of *A. chinensis* ‘Hongyang’ was *de novo* assembled once again. By integrating datasets of HiFi reads, ONT reads, and Hi-C reads, the T2T reference genome of ‘Hongyang’ was initially accomplished and named HongyangV4 [21]. Particularly, HongyangV4 was the first to achieve complete gap-free and haploid-resolved assembly in kiwifruit (Table 1). Compared to HongyangV3, HongyangV4 continued to make a great improvement in genomic integrity and contiguity by filling all the unclosed gaps (Fig. 1). As a result, it obtained ~40 Mb of extra sequences that may harbor previously elusive genes enriched in pathways related to, for example, the plant-type hypersensitive response and specialized metabolism. Furthermore, the sequence and structure of kiwifruit centromeres as well as their monomers (*Ach-CEN153*) were firstly identified and defined, opening a door leading to the characterization of centromere function that was formerly unknown.

The iterative updates of *A. chinensis* ‘Hongyang’ genome have not only reflected the continuous development of sequencing technologies and assembly methods, but also served as the reference for guiding genome assemblies of kiwifruit from the same or closely related species. Accordingly, the progress of the kiwifruit genome assembly can be separated into three distinct phases: draft genome assembly, high-quality genome assembly, and T2T genome assembly (Fig. 1).

**Table 1:** Continued

**Pub Date**	**Genome ID** ^**a**^	**Material name**	**Species name** ^**a**^	**Gender**	**Ploidy**	**Phasing**	**Anchored size**	**Contig N50**	**Gene number** ^**b**^	**Reference**
2025-03-03	JingliW1	wild	*A. henryicallosa*	F	2x	2	662.9/656.8 Mb	19.4/11.1 Mb	42 522/41520	[33]
2025-03-03	KuoyeW2	wild	*A. latifolia*	F	2x	2	610.4/650.0 Mb	21.3/20.5 Mb	42 661/42215	[33]
2025-03-03	MaohuaW2	wild	*A. eriantha*	F	2x	2	647.7/630.4 Mb	20.1/19.1 Mb	42 187/41675	[33]
2025-03-03	MeiweiW3	wild	*A. deliciosa*	F	2x	2	618.9/610.9 Mb	20.6/19.6 Mb	42 453/42290	[33]
2025-03-03	UnknownW1	wild	*A. unknown*	F	2x	2	624.0/628.1 Mb	20.3/18.1 Mb	43 426/42088	[33]
2025-04-01	TianyuanhongV1	Tianyuanhong	*A. arguta*	F	4x	1	693.4 Mb	21.0 Mb	47 899	[34]
2025-06-05	DuieW1	wild	*A. valvata*	F	4x	2	695.8/645.3 Mb	1.6/1.3 Mb	46 646/42275^K^	[35]
2025-07-07	MeiweiW4	wild	*A. deliciosa*	F	6x	6	3910.0 Mb in total	5.9 Mb	249 392 in total	[36]

a The Genome IDs and species names are sourced from the KPGD database. If unavailable, the KPGD naming convention is followed.

b The numerical values are retrieved from their corresponding publications. If unavailable, the values recorded in the KPGD database are used and marked with the capital letter K.

In the ‘draft’ phase, kiwifruit genomes were assembled based only on short reads produced by the next-generation sequencing platforms, such as Illumina HiSeq and Roche 454. This phase typically spanned from 2013 to 2018. In fact, there are only two reported genomes and three different versions during this phase (Fig. 1). While the first kiwifruit genome HongyangV1 was published in 2013 [12], the second genome Red5V1 was not released until 2018 [16]. Notably, the sequencing material of Red5V1 was also selected from *A. chinensis* and is distinctive for its red-fleshed fruit. This study strongly highlighted the importance of manual annotation in improving individual gene models. For instance, a critical *EXPANSIN* gene family involved in fruit softening, which had been missed by automated annotation, was precisely defined and functionally elucidated through manual curation.

In the ‘high-quality’ phase, kiwifruit genomes were assembled based mainly on long reads sequenced by the PacBio Sequel system. This phase primarily spanned from 2019 to 2022 (Fig. 1). Similar to the HongyangV3 genome [18], the WhiteV1 genome was published concurrently in 2019, marking a significant milestone in kiwifruit genomics by providing the high-quality reference for a new species [17]. The availability of the WhiteV1 genome could facilitate comparative sequence analysis between species within the genus *Actinidia*. Accordingly, the divergence time between *A. chinensis* and *A. eriantha* was firstly estimated to be 3.3 million years ago (Mya). Moreover, the sequencing of wild germplasm materials, such as the MaohuaW1 and MeiweiW1 genomes [19, 25], provides a valuable genomic resource for harnessing the potential of untapped genetic diversity to enhance cultivated kiwifruit varieties. Importantly, genes involved in ascorbic acid biosynthesis, fruit skin morphology, and disease resistance have been genome-wide identified, revealing key transcription factors and biosynthetic pathways.

In the ‘T2T’ phase, kiwifruit genomes were highly recommended to be assembled by integrating the HiFi and ONT ultra-long reads. This phase started in 2022 and marks a transformative era in kiwifruit genomics (Fig. 1). In parallel with the T2T-level assembly of the HongyangV4 genome [21], significant progress was also made in *de novo* sequencing and accurately characterizing the physical genomes of many other kiwifruit species and cultivars, including MidaoV1 (*A. eriantha*), DonghongV1 (*A. chinensis*), KuoyeW1 (*A. latifolia*) and so on [22, 24, 34]. These T2T genomes enable a more precise understanding of the complete genetic architecture and previously inaccessible regions, such as telomeres, centromeres, and highly repetitive sequences, which would offer unprecedented insights into their structural variations, genome evolutions, and biological potentials for genetic improvements of kiwifruit. In parallel, the advancement of haplotype-resolved assembly has become pivotal for accurately discerning allelic variations and allele-specific expression (ASE). A compelling demonstration of its power lies in the study of kiwifruit sex chromosomes. Leveraging haplotype-phased T2T genomes of male (XY) plants, researchers can directly resolve and differentiate the X and Y haplotypes [23, 28]. This approach has enabled the precise mapping of sex-determining region (SDR), characterization of allelic diversity between sex chromosomes, and investigation of haplotype-specific expression patterns, thereby providing direct insights into the evolutionary and functional dynamics of dioecy in kiwifruit.

It should be noted that the separation of different phases primarily highlights the advancement in the quality and completeness of genome assembly, rather than representing strict thematic or chronological boundaries. Each phase reflects the ongoing development of sequencing technologies and computational tools, from early draft genomes with fragmented assemblies to high-quality genomes with more accurate representations to recent T2T genomes achieving completely gap-free assemblies. However, while ‘T2T’ marks the end of genome assembly in terms of achieving completeness, it is far from the end of genomics. Instead, it opens up new frontiers for exploration and innovation in the post genome-wide association studies of kiwifruit. These frontiers encompass the functional characterization of genes (genomics), the regulation of their expression (epigenomics and transcriptomics), and the dynamics of their encoded proteins and metabolites (proteomics and metabolomics), each corresponding to a major omics discipline (Table 2).

**Table 2:** An integrated overview of plant multi-omics methodologies and breeding applications

Omics layers	Key Questions	Core technologies	Data analyses	Representative bioinformatics tools	Major applications for trait discovery and breeding
Genomics	What could happen	BGI, Bionano Genomics, Hi-C, Illumina, Oxford Nanopore, PacBio HiFi/Revio, SNP microarray	Allelic variation analysis, Candidate gene prediction, Centromere identification, Comparative genomics, Genome editing, GWAS, Molecular marker development, Pangenome construction, Phylogenetic inference, Population genetics, QTL mapping, Variant calling and annotation (SNPs, InDels, SVs)	ADMIXTURE, BLAST+, Bowtie2, BWA, bcftools, Omega, FastTree, GATK, IQ-TREE, InterProScan, JCVI, MAFFT, MCScanX, MISA, MUMmer, OrthoFinder, Panaroo, PCA, PGGB, Picard, PLINK, PopGenome, quarTeT, RAxML, RepeatMasker, Roary, SAMtools, SnpEff, SSR2Marker, SynVisio, VCFtools, vg, WGCNA	(i) Gene discovery: Identifying genes and QTLs underlying agronomic traits (e.g. yield, stress tolerance, quality); (ii) Molecular breeding: Enabling marker-assisted selection (MAS) and genomic selection; (iii) Germplasm characterization: Assessing genetic diversity, evolution, and identifying novel alleles.
Epigenomics	When it will happen	ATAC-seq, ChIP-seq, CUT&Tag, Hi-C, WGBS	3D interaction analysis, Chromatin accessibility analysis, DMR analysis, Histone modification analysis, Motif enrichment analysis, Peak calling	Bismark, DiffBind, HOMER, MACS2, MEME Suite	(i) Adaptation mechanism: Deciphering epigenetic regulation in stress memory and environmental adaptation; (ii) Gene regulation: Linking epigenetic marks to spatiotemporal gene expression control; (iii) Epibreeding: Developing epigenetic markers for predictive breeding.
Transcriptomics	What will happen	RNA-seq, Iso-seq, scRNA-seq, Spatial transcriptomics	ASE analysis, AS analysis, Cell type identification, DEG analysis, Gene/ncRNA expression profiles, WGCNA	Alevin, CellPhoneDB, Cell Ranger, DESeq2, edgeR, FeatureCounts, HISAT2, Kallisto, Limma, Monocle, PAGA, PseudoOrder, RSEM, Salmon, Scanny, Seurat, STAR, StringTie, TBtools, Trinity, Velocyto	(i) Regulatory networks: Uncovering co-expression modules and key regulators in development and stress responses; (ii) Mechanistic insights: Identifying candidate genes and signaling pathways; (iii) Cellular heterogeneity: Resolving cell-type-specific responses and developmental trajectories.
Proteomics	What is happening	2D-GE, DIA, Label-Free, LC–MS/MS (Shotgun, SRM/PRM), SILAC, SWATH-MS, TMT/iTRAQ	Differential expression analysis, Functional enrichment and pathway analysis, Peptide/Protein identification, PPI prediction, Protein structure/function prediction, PTM site localization, Quantification	AlphaFold, Comet, Cytoscape, DAVID, DIA-NN, Enrichr, GeneMANIA, GProX, IPA, MaxQuant, MASCOT, MetaMorpheus, MSFragger, Perseus, Proteome Discoverer, Scaffold, Sequest, Spectronaut, STRING	(i) Functional annotation: Identifying and quantifying active proteins and critical PTMs (e.g. phosphorylation); (ii) Stress physiology: Understanding proteome-level adaptation to biotic and abiotic stresses; (iii) Biomarker development: Discovering protein biomarkers for early-stage trait selection.
Metabolomics	What happened	CE-MS, DART-MS, GC–MS, IMS-MS, LC–MS, MALDI imaging, NMR spectroscopy, Targeted/Untargeted profiling	Cluster analysis, Correlation network analysis, Differential analysis, Flux estimation, Metabolite identification, Metabolic pathway analysis, Multivariate statistical validation, OPLS-DA, Peak alignment and normalization, PCA, PLS-DA	ASICS, ChemStation, Compound Discoverer, CytoScape, GNPS, MESA, MestReNova, MetaBoAnalyst, MS-DIAL, mzMine, Progenesis QI, SIMCA, Skyline, XCMS	(i) Biochemical phenotyping: Linking genotype to the final biochemical output (e.g. flavor, nutrition, defense); (ii) Trait enhancement: Identifying key metabolites for quality improvement and metabolic engineering; (iii) Systems biology: Providing a functional readout of cellular status under different conditions.

(Continued)

**Table 2:** Continued

Omics layers	Key Questions	Core technologies	Data analyses	Representative bioinformatics tools	Major applications for trait discovery and breeding
Interactomics	How it happens	AP-MS, BiFC, Co-IP/MS, FLIM-FRET, LCI, Phage display, Y2H screening	Dynamic/context-specific network analysis, Functional enrichment analysis of network modules, Identification of network hubs/modules/key drivers, PPI network construction, Topological parameter calculation	CentiScaPe, ClusterONE, Cytoscape, CytoHubba, Gephi, GeneMANIA, HitPredict, IntAct, MCODE, networkAnalyst, PSPC, STRING	(i) Pathway elucidation: Mapping signaling cascades and metabolic protein complexes; (ii) Host-pathogen interactions: Decoding molecular battles (e.g. pathogen effector vs. host resistance protein networks); (iii) Target prioritization: Identifying critical interaction nodes for genetic manipulation or chemical intervention.
Phenomics	What it looks like	High-throughput phenotyping (RGB, Hyperspectral, Fluorescence, Thermal imaging), IoT platforms, LiDAR, Sensor networks, UAV-based field phenotyping	Analysis of high-dimensional phenotypic data, Genotype–phenotype modeling, Image processing, segmentation and feature extraction, Multi-sensor/multi-modal data fusion, Time-series growth dynamics analysis, Trait ontology and standardization	3DField, BreedVision, CropQuant, DIRT, DJI Terra, ENVI, Field Scanalyzer, GPRuler, HTpheno, ImageJ / Fiji, LemnaTec, MATLAB, OpenCV, PlantCV, QGIS, R (phenotypic analysis packages), RhizoVision, RootNav, Weka	(i) Large-scale trait measurement: Automated quantification of growth, architecture, and stress responses; (ii) Bridging scales: Integrating molecular omics data with organismal-level phenotypes; (iii) Accelerated breeding: Powering selection decisions in genomic-assisted breeding programs.

### Transcriptome profiling and analysis

**Figure 2:**
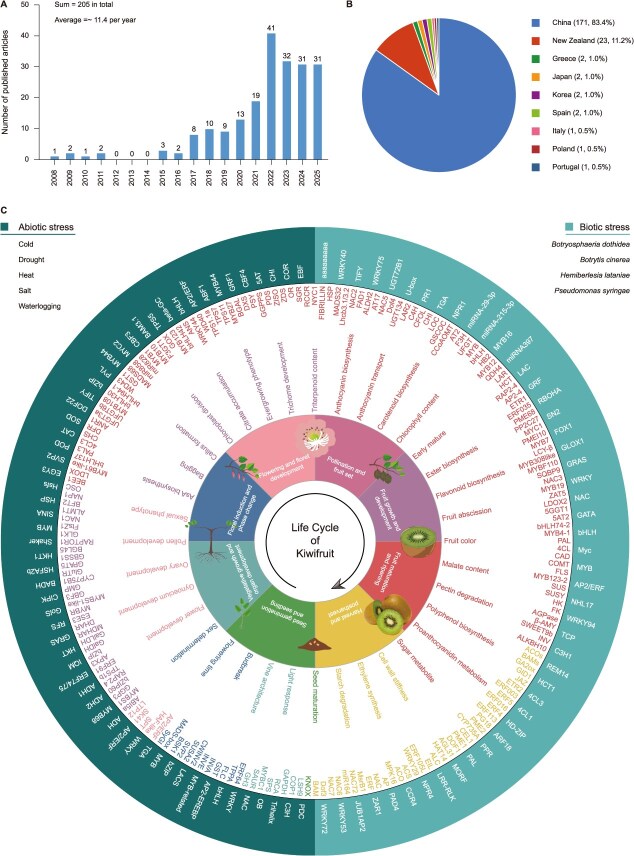
Overview of kiwifruit transcriptomics studies and their trait focuses. (A) Temporal distribution of over 200 published transcriptomic studies, as of 28 August 2025. (B) Geographical distribution of contributing countries, highlighting China's leading contribution (83.4%). (C) A multi-layered circular profile of curated transcriptomic data. The innermost tier delineates the eight key developmental stages (from germination to postharvest). The second tier categorizes the major research focuses within each stage. The third layer visually catalogs all individual genes reported for each stage. The outermost band details stress-related genes, bifurcated into abiotic stress tolerance (left semicircle) and biotic stress resistance (right semicircle).

The transcriptomic studies utilizing RNA sequencing (RNA-seq), expressed sequence tag (EST) profiling, and microarray technologies provide powerful tools for elucidating the genetic mechanisms underlying development, ripening, post-harvest, and stress responses. Unlike the relatively static genomic DNA, transcriptome can exhibit remarkable plasticity and rapid change in RNA abundance when responding to cellular and environmental cues. In kiwifruit, the earliest transcriptomic studies predated the first release of the reference genome and began in 2008 with the advent of EST technology (Fig. 2A and Table S1). By collecting nearly all tissue samples from *A. chinensis*, *A. deliciosa*, *A. eriantha*, and *A. arguta*, a total of 132 577 ESTs were obtained and characterized [37]. Despite the technical challenges posed by the lack of a genomic framework, this groundbreaking work provided the first comprehensive snapshot of the kiwifruit transcriptome, serving as a critical resource for systematic identification of functional genes involved in vitamin C biosynthesis [38], carotenoid accumulation [39], branch development [40], and bud dormancy and flowering [41]. Meanwhile, microarray technology has enabled parallel interrogation of large-scale genomic DNA libraries, such as those conducted on the bud dormancy and ripening process in kiwifruit [42, 43].

Compared to EST and microarray technologies, RNA-seq has demonstrated significantly faster development and broader utilization due to its higher resolution and throughput, laying the foundation for subsequent genomic studies to identify, quantify, and compare the complete set of RNAs within the given cells or tissues. To date (as of 28 August 2025), over two hundred transcriptomic studies encompassing thousands of individual assays have been published on kiwifruit (Fig. 2A and Table S1), covering diverse genotypes [44, 45], developmental processes [46, 47], tissue types [48–50], sexual phenotypes [51, 52], metabolic adaptations [46, 53–55], stress responses [56, 57], and experimental treatments [58–60]. Statistical data indicate that these studies were conducted across nine countries, including China, New Zealand, Greece, Japan, Korea, Spain, Italy, Poland, and Portugal (Fig. 2B and Table S1). Among them, China contributed the highest number of publications on kiwifruit transcriptomics, accounting for 83.4% of the total (Fig. 2B). Further analysis reveals that the research institutions in China involved in these efforts are distributed across 22 provinces, autonomous regions, or municipalities, with Henan, Sichuan, Hubei, and Jiangxi being the most active regions (Table S2). Notably, this distribution closely aligns with the natural geographic distribution of kiwifruit within China, reflecting a synergistic cycle where scientific inquiry addresses local agricultural needs, and in turn, practical challenges from the field inform and enrich the research agenda, jointly powering rural revitalization.

Leveraging the compiled transcriptomic landscape, we undertook a functional excavation and synthesis of this extensive dataset. First, we systematically categorized all reported genes according to the complete growth cycle of kiwifruit, spanning from seed germination and vegetative growth through reproductive development and fruit maturation to postharvest stage, thereby constructing stage-specific gene expression atlases (Fig. 2C). Next, we focused on stress response mechanisms to systematically identify key genes involved in biotic stress resistance (e.g. against canker and soft rot) and abiotic stress tolerance (e.g. mediating responses to cold, drought, and salinity) (Fig. 2C). Collectively, this integrative analysis not only reveals core transcriptional regulatory modules active at different developmental stages but also provides a comprehensive, organized, and searchable gene resource library for molecular breeding in kiwifruit. It is worth noting that, according to our data compilation, the most extensively researched areas have been fruit flesh color and bacterial canker resistance.

Beyond quantifying gene expression levels, transcriptome analysis can also detect alternative splicing variants, which are critical for accurate gene model prediction and annotation in the process of *de novo* genome assembly (Table 2). As presented in the previous section, integration of the latest EST and RNA-seq datasets has refined the genome annotation of *A. chinensis* ‘Hongyang’ to 39 761 protein-coding genes, of which 8245 (20.7%) exhibit multiple transcript isoforms [15], highlighting the extensive alternative splicing landscape in kiwifruit. Additionally, RNA-seq data have facilitated the exploration of candidate genes specific to the SDR of Y chromosome in the ‘Russell’ genome [20]. Furthermore, RNA-seq data enabled precise annotation of centromeric genes in the T2T genome assembly, overcoming challenges posed by their embedded location within the highly repetitive sequences [21].

Another pivotal advantage of transcriptomics lies in its integration with other omics approaches, providing a critical bridge to decipher biological complexity at the system level. Collectively, integrative analysis of transcriptome and metabolome has emerged as the predominant multi-omics strategy, enabling causal inference between gene regulation and metabolic phenotypes. In kiwifruit, this strategy has been extensively employed to pinpoint the genetic determinants linking to early mature trait [61], flesh color change [62], flavor formation [63], flavonoid accumulation [50], aromatic amino acid production [64], salt tolerance [65], and freezing tolerance [66]. Similarly, transcriptome–proteome co-analysis has successfully revealed the key role of *MYB16* in the response of kiwifruit to *Pseudomonas syringae* pv. *actinidiae* (*Psa*) infection [67]. Moreover, the integration of transcriptome and methylationome has demonstrated that *ALKBH10* plays a more substantial role than *ECT9* in modulating fruit quality attributes through m6A-dependent regulation of ripening-related genes in kiwifruit [68].

### Non-coding RNA identification and classification

Non-coding RNAs (ncRNAs), including microRNAs (miRNAs), long non-coding RNAs (lncRNAs), and circular RNAs (circRNAs), are emerging as critical regulators of gene expression at the post-transcriptional level. Similar to coding RNAs, ncRNAs can also be identified and quantified using next-generation sequencing technologies (Table 2). Actually, these technologies can capture the entire transcripts, enabling the simultaneous detection of both coding RNA and ncRNA. By re-analyzing the data of nine RNA-seq libraries, a total of 7051 lncRNAs have been identified from *A. chinensis* ‘Hongyang’, providing the first comprehensive catalog of these molecules in kiwifruit [69]. Likewise, circRNAs and lncRNAs that are particularly associated with the species-specific response to *Psa* infection were comparatively detected and characterized in four kiwifruit materials [57, 70]. Additionally, a computational approach has been introduced to annotate conserved miRNAs from publicly available genomic sequences in kiwifruit, resulting in 16 miRNAs from *A. chinensis*, 3 from *A. eriantha* and 1 from *A. delicosa* [71].

In contrast, by using specialized library preparation protocols, RNA-seq can be tailored to enrich for specific ncRNA classes, which would effectively reduce noise and enhance accuracy. Based on this, ncRNA sequencing studies have revealed the presence of numerous sequences that play pivotal roles in diverse growth and developmental processes as well as various biotic and abiotic stress responses. For instance, deep sequencing of small RNA libraries was adopted to identify both conserved and novel miRNAs in ripe fruits of *A. deliciosa* ‘Hayward’ [72]. Due to the synergistic relationship, coding RNA and ncRNA sequencing data can effectively complement each other for unraveling their functional roles in complex biological systems at both transcriptional and post-transcriptional levels. By integrating the expression of coding and non-coding sequences, miR164 has been determined to regulate fruit ripening through inhibition of NAC transcription factors in kiwifruit [72]. Similarly, the construction of miR482-NBS-LRR regulatory network reveals how miRNAs enhance resistance to *Psa* infection in kiwifruit [73]. Furthermore, multiple lncRNAs have also been shown to potentially modulate gene expression networks during fruit ripening and under disease conditions [74–76]. Specially, while transcriptomic studies provide a broad overview of gene expression changes, ncRNA identification and characterization offer insights into the regulatory layers that fine-tune these changes.

### Proteome characterization and quantification

Proteomics, the large-scale study of proteins, has emerged as a powerful tool for characterizing and quantifying the dynamics of proteins that are closely related to gene expression. In kiwifruit, proteomic studies have made significant progress in recent years, providing valuable insights into plant growth and development, fruit maturation and ripening, post-harvest physiology and storage, and abiotic and biotic stress tolerance (Table 3). By combining SDS-PAGE and 2-DE analysis, protein patterns of fruit, seed, and leaf were compared in *A. deliciosa*, and the highest number of protein bands were found in fruit [77]. Likewise, comparative proteomic study was performed to identify biomolecular markers elicited by Cr treatment on germinating kiwifruit pollen [78]. Using 1D-SDS-PAGE and mass spectrometry, 102 differentially represented proteins participating in energy, defense, and cell structure were identified during post-harvest ripening of ozone-treated fruits [92]. Moreover, those ripening-related genes in ozone-treated fruits were further selected for expression quantification by RT-qPCR, achieving multi-omics association analysis to uncover the complex interactions between transcriptomic and proteomic data in kiwifruit ripening physiology [93]. As an efficient approach, proteomics has also been used to elucidate the biochemical changes of kiwifruits that were treated with ABA, ethylene and chilling [94, 96–98, 100].

**Table 3:** A compendium of foundational kiwifruit proteomic and metabolomic studies

**Research focus and study objective**	**Tissue type/Developmental phase**	**Materials**	**Methodology**	**Reference**
Plant growth and development				
Protein patterns of diverse organs	Leaves, fruits and seeds	*A. deliciosa*	Proteome	[77]
Proteomic changes under Cr treatment	Pollen	*A. deliciosa*	Proteome	[78]
Metabolic profiles among different species	Ripe fruits	A group of species	Metabolome	[79]
Metabolic profiles between different germplasms	Normal fruit and early mature fruit	*A. eriantha*	Metabolome	[61]
Metabolic changes under salt treatment	Leaves and roots	*A. valvata* and *A. deliciosa*	Metabolome	[65]
Metabolic profiles among different cultivars	Ripe fruits	*A. eriantha*	Metabolome	[80]
Metabolic changes under bagging treatment	Pre-ripe fruit	*A. eriantha*	Metabolome	[81]
Metabolic changes under forchlorfenuron treatment	Fruits at five developmental stages	*A. deliciosa*	Metabolome	[82]
Metabolic changes under bagging treatment	Fruits at five developmental stages	*A. chinensis*	Metabolome	[83]
Identification of specific medicinal components	Fruits and roots	A group of species	Metabolome	[50]
Differential accumulation of flavonoids among organs	Roots, stems, leaves, and fruits	*A. chinensis*	Metabolome	[84]
Metabolic profiles across diverse tissues	Flowers and different parts of the fruit (skin, outer pericarp, inner pericarp, and core).	*A. chinensis*	Metabolome	[85]
Fruit maturation and ripening				
Metabolic changes during fruit development	Fruits at six developmental stages	*A. deliciosa*	Metabolome	[86]
Nutritional component dynamics during fruit development	Fruits at five developmental stages	*A. chinensis*	Metabolome	[87]
Anthocyanin profiles among different species	Ripe fruits	*A. arguta* and *A. chinensis*	Metabolome	[88]
Organic acid dynamics during fruit development	Fruits at seven developmental stages	*A. eriantha*	Metabolome	[89]
Metabolic changes during fruit development	Fruits at 11 developmental stages	*A. chinensis*	Metabolome	[90]
Flavonoid and carotenoid changes during fruit development	Fruits at five developmental stages	*A. chinensis*	Metabolome	[62]
Anthocyanin changes during fruit development	Fruits at four ripening stages	*A. arguta*	Metabolome	[91]
Post-harvest physiology and storage				
Proteomic changes under ozone treatment	Harvested fruits	*A. deliciosa*	Proteome	[92]
Proteomic changes under ozone and sodium nitroprusside treatments	Harvested fruits	*A. deliciosa*	Proteome	[93]
Proteomic changes under ethylene and chilling treatments	Harvested fruits	*A. deliciosa*	Proteome	[94]
Metabolic changes during fruit postharvest ripening	Fruits at seven ripening stages	*A. deliciosa*	Metabolome	[95]
Proteomic changes under ABA treatment	Wound-induced suberization in harvested fruits	*A. deliciosa*	Proteome	[96]
Proteomic changes under chilling treatment	Harvested fruits	*A. deliciosa*	Proteome	[97]
Proteomic changes under ethylene treatment	Harvested fruits	*A. deliciosa*	Proteome	[98]
Metabolic changes during fruit postharvest ripening	Fruits at five ripening stages	*A. deliciosa*	Metabolome	[99]
Proteomic changes under chilling treatment	Harvested fruits	*A. arguta*	Proteome	[100]
Metabolic changes under wounding treatment	Ripe fruits	*A. deliciosa*	Metabolome	[101]
Metabolic changes under mechanical treatment	Ripe fruits	*A. deliciosa*	Metabolome	[102]
Metabolic changes during fruit development	Fruits at six developmental stages	*A. chinensis*	Metabolome	[103]
Metabolic changes under ethylene and 1-MCP treatments	Ripe fruits	*A. chinensis*	Metabolome	[104]

(Continued)

**Table 3:** Continued

**Research focus and study objective**	**Tissue type/Developmental phase**	**Materials**	**Methodology**	**Reference**
Metabolic changes under chitosan treatment	Ripe fruits	*A. chinensis*	Metabolome	[105]
Metabolic changes under chilling treatment	Ripe fruits	*A. chinensis*	Metabolome	[106]
Metabolic changes under brassinolide treatment	Ripe fruits	*A. deliciosa*	Metabolome	[107]
Abiotic and biotic stress tolerance				
Proteomic changes following *Psa* infection	Shoots	*A. chinensis*	Proteome	[108]
Proteomic changes following *Psa* infection	Leaves	*A. deliciosa*	Proteome	[109]
Proteomic changes following *Botrytis cinerea* infection	Fruits	*A. deliciosa*	Proteome	[110]
Metabolic changes following *Psa* infection	Leaves and stems	*A. arguta* and *A. deliciosa*	Metabolome	[111]
Metabolic changes under chilling treatment	Shoots	*A. arguta*	Metabolome	[66]
Proteomic changes following *Psa* infection	Leaves	*A. deliciosa*	Proteome	[67]
Metabolic changes among species following *Psa* infection	Shoots	*A. arguta* and *A. deliciosa*	Metabolome	[112]
Metabolic changes under auxin treatment	Healthy fruits and *Botrytis cinerea*-infected fruits	*A. chinensis*	Metabolome	[113]
Proteomic changes under salicylic acid treatments	Healthy leaves and *Psa*-infected leaves	*A. deliciosa*	Proteome	[114]

To delineate the defense mechanisms against *Psa* infection, 117 and 58 differentially represented proteins were respectively identified in artificially inoculated shoots and leaves through a combination of 2-DE, nanoLC-ESI-LIT-MS/MS, and qPCR procedures, which have promoted the development of novel bioassays for pathogen detection in kiwifruit [108, 109]. Furthermore, a total of 1681 differentially expressed proteins were identified between the susceptible cultivar *A. chinensis* ‘Hongyang’ and the resistant cultivar *A. deliciosa* ‘Jinkui’ after *Psa* infection [67]. For developing effective strategies to control kiwifruit bacterial cankers, 4D proteomics was used to decipherer the potential mechanisms of SA in promoting *Psa* resistance [114]. As a result, numerous resistance-related proteins, including phenylpropanoid biosynthesis and MAPK cascade, were upregulated by SA treatment, providing a theoretical basis for the use of SA in kiwifruit production. In another study, 292 kiwifruit proteins were identified as associated with the response to *Botrytis cinerea*, the causal agent of gray mold and the most significant post-harvest disease in kiwifruit industry [110].

### Metabolome examination and measurement

Metabolites, small molecules (molecular masses ≤1500 Da) produced through all the biochemical reactions within a cell, tissue or organ, are directly responsible for various observable phenotypes, such as fruit quality, texture changes, nutritional composition, stress response, and environmental adaptation. Metabolomics serves as an effective approach for the comprehensive detection and quantification of metabolites on a global scale [115]. In kiwifruit, progress in understanding the key biological processes mentioned above, which span plant growth and development, fruit maturation and ripening, postharvest physiology, and stress tolerance, has also been significantly driven by advancements in metabolomic technologies (Table 3).

Firstly, metabolomics has been widely used to study the metabolic changes that occur during kiwifruit development [86, 87, 89], ripening [62, 90], and postharvest storage [95, 99, 103], expanding our understanding of the biochemical basis of fruit quality and nutritional value. For example, over 500 metabolites categorizing into 10 distinct modules were comprehensively identified at 11 different developmental and ripening stages, forming a high-resolution metabolic regulatory network in kiwifruit [90].

Secondly, metabolomics has been instrumental in characterizing the composition and content differences of key metabolites among the major kiwifruit tissues, such as fruit skin, outer pericarp, inner pericarp, fruit core, root, stem, and leaf, providing critical insights into their unique biochemical properties and potential utilization values in kiwifruit industry [50, 84, 85]. Particularly, due to the significant medicinal properties of kiwifruit root, a comparative metabolomic analysis was conducted between the root and fruit tissues, which suggested that flavonol and phloretin may act as bioactive components in kiwifruit roots and contribute to their widespread use in traditional Chinese medicine [50].

Thirdly, metabolomic studies have revealed significant differences in metabolite profiles among different kiwifruit species, cultivars, and varieties, shedding light on their distinctive characteristics on biochemical compositions, nutritional qualities, biosynthetic pathways, regulatory mechanisms, and adaptive responses to environmental conditions. Using a combination of NMR spectroscopy and fluorescence measurement, 41 more significantly different metabolites were found in *A. arguta*, *A. deliciosa*, and *A. eriantha*, demonstrating their genetic diversity after kiwifruit species differentiation [79]. By integrating comparative transcriptomic analysis, many functional genes associated with the biosynthesis and regulation of key metabolites were successfully identified and characterized [61, 80, 88, 91]. Notably, the MYB10 and HLH5 transcription factors have been identified as key regulators involved in the pigmentation of red-fleshed *A. chinensis* ‘Hongyang’, while F3H, F3GT, and MYB110 are likely to play crucial roles in the pigmentation of purple-fleshed *A. arguta* ‘Mini Amethyst’ [88]. Furthermore, another study revealed that six MYB, six bHLH, and one WDR transcription factors were significantly associated with cyanidin-3-O-galactoside, which is responsible for the red coloration in a full red-type *A. arguta* ‘Jinhongguan’ [91].

Finally, metabolomic analyses have provided invaluable insights into how kiwifruit responds to a wide variety of stresses and treatments, including pathogen infections [111–113], growth regulators (ethylene, 1-MCP, auxin, brassinolide, forchlorfenuron) [82, 104, 107, 113], chitosan coating [105], temperature fluctuations [66, 106], salinity [65], wounding [101], compression [102], and bagging [81, 83]. Specifically, the complex remodeling of kiwifruit response to *Psa* infection has been explored, which could be applicable for guidance in the development of innovative disease management strategies [111, 112].

### Methylome exploitation and reprogramming

Methylomics, the study of methylation patterns and their functional roles, has emerged as a powerful tool for understanding the epigenetic regulation of gene expression. Recently, an increasing number of studies are being conducted to uncover how methylation changes influence the processes of kiwifruit development and ripening [68, 116]. By using LC–MS/MS and MeRIP sequencing technologies, the levels of N6-methyladenosine methylation` (m6A) were quantified across four key stages, from early development through to ripening, displaying a gradual decrease and exhibiting an inverse correlation with the expression of ripening-related genes [68]. In another study, gene co-expression networks were constructed based on comprehensive expression datasets of kiwifruit, which strongly indicated that m6A-related genes are important regulators of fruit development and ripening [116]. Meanwhile, these networks also showed that m6A modifications are central to the modulation of various biotic and abiotic stress responses.

Beyond RNA methylation, DNA methylation represents another critical epigenetic modification. However, research focusing on DNA methylation in kiwifruit remains relatively limited. To date, only one study has quantified the level of DNA methylation at the genomic level and investigated its roles in climate change adaptation through epigenetic modifications [117]. Another related study is a genome-wide identification of DNA methylases and demethylases that determine DNA methylation pattern by dynamically adding and removing methyl groups from cytosine residues [118]. As a result, nine methylases and seven demethylases were comprehensively identified and characterized, which offers valuable insights into the complexity and composition of these two gene families in kiwifruit.

## Genomic resources: from raw data to knowledge bases

Genomic resources for kiwifruit (*Actinidia* spp.) have been scattered across multiple repositories, but most are inconsistent, fragmented, and incomplete. As of 18 September 2025, the NCBI database (https://www.ncbi.nlm.nih.gov/datasets/genome/) houses 13 kiwifruit genome assemblies, while a search for ‘*Actinidia*’ in the Genome Warehouse (GWH, https://ngdc.cncb.ac.cn/gwh) returns 64 records, of which 35 are genome projects (Table 4). Other platforms provide even more limited coverage: EnsemblPlants (https://plants.ensembl.org/index.html) includes only a single reference genome (*A. chinensis* ‘Red5’), and the Plant Garden database (https://plantgarden.jp/en/index) in Japan hosts genome assemblies for five kiwifruit species. Given this, the fragmentation and exponential growth of kiwifruit genomic data highlights the need for a dedicated database, particularly with advanced functional and analytical capabilities, to centralize and integrate these dispersed resources for the entire genus.

**Table 4:** Comparative overview of integrated multi-omics resources for kiwifruit

**Feature summary**	**KPGD**	**KGD**	**KIR**	**NCBI**	**GWH**	**EnsemblPlants**	**GERDH**	**eFP**	**kfALP**
Resource focus	Specialized	Specialized	Specialized	General	General	General	General	Specialized	Specialized
Resource type									
Released genomes	33	4	1	13	35	1	–	–	–
Haplotype-resolved	55	0	0	0	49	0	–	–	–
Structural annotations	55	4	1	5	10	1	–	–	–
Functional annotations	55	4	1	0	0	0	–	–	–
Gene-based pangenome	Analyzed	–	–	–	–	–	–	–	–
Transcriptome data	Curated	Curated	–	Distributed	Distributed	–	Curated	Curated	–
Expression profiles	Count/FPKM matrices	–	–	–	–	–	TPM matrices	Heatmaps/Matrices	–
Proteome data	Curated	–	–	–	–	–	–	–	Curated
Metabolome data	Curated	–	–	–	–	–	–	–	–
Online tools									
Genome browser	Integrated	Integrated	Integrated	Integrated	–	–	–	–	–
BLAST program	Integrated	Integrated	Integrated	Integrated	Integrated	Integrated	Integrated	–	–
CRISPR design	Provided	–	–	–	–	–	–	–	–
SV detection	Provided	–	–	–	–	–	–	–	–
ID converter	Provided	Provided	–	–	–	–	–	–	–
GO/KEGG enrichment analysis	Provided	–	–	–	–	–	Provided	–	–
DGE/WGCNA analysis	Provided	–	–	–	–	–	Provided	–	–
Access (URL)	https://kiwifruitgenome.atcgn.com/	https://kiwifruitgenome.org/	http://kir.atcgn.com/	https://www.ncbi.nlm.nih.gov/	https://ngdc.cncb.ac.cn/gwh/	https://plants.ensembl.org/	https://dphdatabase.com/	https://bar.utoronto.ca/efp_actinidia/	Not available

In kiwifruit, large volumes of omics data have been generated since the first reference genome released in 2013 [12]. Aligning with the triphasic advancement in kiwifruit genome research, the associated databases have also progressed through three distinct developmental stages (Fig. 1). This transformation is clearly demonstrated by the rapid expansion of genome-anchored multi-omics data across the three database versions: Kiwifruit Information Resource (KIR) [15], Kiwifruit Genome Database (KGD) [119], and Kiwifruit PanGenome Database (KPGD) [13]. While KIR was initially built with only a single reference genome, KGD comprised four genomes available at the time of its establishment, and KPGD currently hosts as many as 33 genomes represented by a total of 55 haplotype-resolved assemblies (Table 4). Concurrently, they have substantially expanded their omics data repertoire to include transcriptomic, proteomic, metabolomic, epigenomic, and genome resequencing datasets during each database development cycle. Complemented by multiple visualization applications and web tools, such as CRISPR design, SV detection, and GO/KEGG enrichment analysis, the latest-generation database extends beyond a conventional genomic repository to serve as a comprehensive platform for the kiwifruit research and breeding community [13].

Complementing genome databases, a number of systematic studies dedicated to specialized topics have also established high-quality resources and conceptual frameworks for kiwifruit science (Table 4). For example, the Gene Expression Regulation Database of Horticultural Plants (GERDH, https://dphdatabase.com/) offers gene expression data for a few kiwifruit species [120]. In addition, a customized Electronic Fluorescent Pictograph (eFP, https://bar.utoronto.ca/efp_actinidia/cgi-bin/efpWeb.cgi) browser was developed to visualize spatiotemporal gene expression profiles of different tissue types and developmental stages from *A. chinensis*, with functionally integrated modules for genome-wide transcription factor prediction and weighted gene co-expression network analysis [121]. Moreover, the kfALP resource was systematically constructed to catalog amyloplast-localized proteins, offering novel mechanistic insights into amyloplast biogenesis and differentiation across kiwifruit cultivars [122]. Collectively, these specialized resources constitute indispensable complements to genome databases within the kiwifruit research community.

## Genetic diversity: from sequence variation to phenotypic consequences

### High-density linkage maps

Prior to genome assembly, high-density genetic linkage maps serve as a cornerstone of modern plant genetics in providing critical frameworks to investigate and decipher the inheritance mechanisms of complex agronomic traits across parental lines. As summarized in Table 5, the inaugural genetic map study of kiwifruit commenced in 2000 by using a combination of SSR and AFLP markers through the pseudo-testcross mapping strategy [123]. Notably, this study generated two linkage maps: a female map comprising 71 SSR and 89 AFLP markers, and a male map containing 28 SSR and 87 AFLP markers along with one sex-determinant locus that was successfully localized on the linkage group no. 25. On top of that, a higher-density genetic map was constructed with 644 SSR markers, which delineated 29 linkage groups corresponding to the chromosome number of kiwifruit haploid genome [124]. Another significant breakthrough was the fine mapping of sex-determinant locus to a subtelomeric region on the Y chromosome, supporting the observation of recombination inhibition based on cytological examination [138].

**Table 5:** Major advances from genetic studies of kiwifruit

**Population**	**Study objective**	**Population size**	**Marker type**	**Marker number**	**Identified genes/loci/markers**	**Reference**
*A. chinensis* and *A. callosa*	Linkage map construction	94	SSR and AFLP	160 for female and 116 for male	The sex locus on Chr25	[123]
*A. chinensis*	Linkage map construction and SDR identification	272	SSR	644	The sex locus on Chr25	[124]
*A. chinensis*	Mapping of resistance and defence genes against *Psa*	272	SSR and InDel	71	LRR receptor-like serine/threonine-protein kinase FLS2	[125]
*A. chinensis*	Genome quality improvement and molecular marker development	94	SNP	12 596	SmX, Ke225, UDK096, and SmY1	[126]
*A. rufa* and *A. chinensis*	Development of molecular markers for sex identification	174	SNP	2426 for female and 4214 for male	Three sex-specific SSR markers on Chr25	[127]
*A. chinensis*, *A. deliciosa* and *A. eriantha*	Identification of the locus for ascorbic acid biosynthesis	80	SNP	\	qAsA26.1	[128]
*A. chinensis*	Identification of the locus conferring *Psa* resistance	236	SNP	9875 for female and 9327 for male	Achrdv1x03g030950 (MADS-box protein AGL42), Achrdv1x08g088570 (Ammonium transporter 2), Achrdv1x15g160370 (Alpha-glucan phosphorylase)	[129]
*A. chinensis*	Identification of the locus conferring *Psa* resistance	235	SNP	39 322	Receptor-like kinase	[130]
*A. eriantha*	Identification of the locus controlling flower- and leaf-related traits	143	SNP	946 337	Eleven loci	[131]
*A. deliciosa*	Identification of the locus controlling king flower number, fruit number and weight, dry matter accumulation, and storage firmness	268	SNP	3686 for female and 3940 for male	Nine QTLs	[132]
*A. arguta*	Development of a 135 K SNP genotyping array	40	SNP	134 729	The sex locus on Chr03	[133]
*A. eriantha*	Identification of the locus controlling fruit quality and yield	140	SNP	~8 880 000	WUSCHEL, CDK1, AO1 and CO1	[134]
*A. eriantha*	Identification of the locus controlling fruit shape-related traits	216	SNP	1 790 395	F-box, MADS4, WOX, OVATE	[135]
*A. chinensis*	Identification of the locus controlling trunk and branch diameter growth	173	SNP	6506	Achhyv3x14g174910 (Heavy metal-associated isoprenylated plant protein), Achhyv3x29g353810 (EIN3-binding F-box protein)	[136]
*A. arguta*	Identification of the locus underlying key fruit quality traits	315	SNP	134 729	MYB110	[137]

Subsequently, advances in high-throughput sequencing technologies have significantly enhanced the efficiency of SNP detection and utilization, enabling the construction of high-density genetic linkage maps with unprecedented precision (Table 5). Using genome-wide SNP markers from 12 586 restriction-site-associated DNA (RAD) loci, a high-density SNP-based genetic map was firstly constructed in *A. chinensis* [126]. Meanwhile, 6347 and 6470 informative markers were developed for the female and male parents, respectively. Moreover, the resulting map has placed about 120 Mb unanchored sequences and corrected some mis-joined scaffolds of the HongyangV1 genome assembly.

Over the past decade, the expanding availability of high-quality genome assemblies and whole-genome sequencing data has greatly facilitated the construction of high-density genetic linkage maps with multiple types of DNA markers, providing a robust framework for fine mapping and molecular dissection of genomic regions associated with complex traits in kiwifruit (Table 5). For instance, sex-linked SNP and SSR markers were identified in the SDR through RAD-seq using an interspecific F1 segregating population between *A. rufa* and *A. chinensis*, which can be used to distinguish male and female vines [127]. Using a tetraploid F1 population, sex-linked SNP markers were all mapped to Chr03 of the reference genome, revealing a single major-effect sex-determination locus on linkage group 3 (LG3) in *A. arguta* [133].

As most traits analyzed in linkage mapping populations exhibit quantitative inheritance, their associated loci are typically distributed across multiple linkage groups. Using an F1 population derived from diploid *A. chinensis*, quantitative trait locus (QTL) mapping has detected one major-effect locus on LG27 and six minor-effect loci on LG3, LG14, LG15, LG22, LG24, and LG28 for *Psa* resistance, demonstrating the polygenic architecture of this trait [129]. Similarly, four significant QTLs conferring resistance to *Psa* were consistently identified on distinct linkage groups (LG1, LG2, LG4, and LG7) in a tetraploid *A. chinensis* population, with each explaining 9.7% to 12.8% of the phenotypic variance [130]. Following these discoveries, genome annotation coupled with expression profiling has pinpointed several promising genes within these loci, such as MADS-box protein and Ammonium transporter, which are hypothesized to be responsible for the resistance phenotype (Table 5).

In addition to gender determination and pathogen resistance, high-resolution QTL mapping has also been extensively utilized to dissect the genetic architecture of numerous agronomically important traits in kiwifruit, including trunk diameter, branch diameter, bud burst, flower number, flowering time, fruit number, fruit weight, flesh color, flesh acidity, sugar content, fruit size, and vitamin C content [125, 128, 132, 136, 137]. Through this approach, key candidate genes underlying these traits have been uncovered, ranging from transcriptional regulators to enzymes. Notable examples include F-box, MADS4, WOX, OVATE, WUSCHEL, CDK1, AO1, and CO1 (Table 5). Particularly, a conserved *MYB* gene, known to regulate anthocyanin biosynthesis in *A. chinensis* [139], was pinpointed to a major QTL through high-density genetic mapping in *A. arguta* [137].

### Genome-wide association study

Genome-wide association study (GWAS) is another effective approach for linking genetic variation to phenotypic diversity. While QTL mapping utilizes structured populations with limited recombination events, GWAS could leverage historical recombination accumulated in natural populations over generations. Thus, GWAS provides opportunities to discover the genomic signatures underlying kiwifruit evolution and domestication by characterizing linkage disequilibrium (LD) decay patterns (Table 5). For example, GWAS using RAD sequencing data from 143 male kiwifruit germplasms of *A. eriantha* has identified association signals at various loci for a total of 11 flower and leaf traits, providing theoretical basis for the breeding of elite pollinator cultivars [131]. Additionally, population structure analysis further resolved these germplasms into two major groups that correlate strongly with their geographic origins, offering insights into the diversity patterns and evolutionary processes within the genus *Actinidia*.

In parallel, the GWAS approach has proven equally valuable for dissecting the genetic architecture of multiple fruit traits, such as the quality, yield, and sharp (Table 5). By using the resequencing technology on 140 female individuals, GWAS enables simultaneous identification of 59 genomic regions significantly associated with 8 key agronomic traits in *A. eriantha* [134]. Notably, this study makes an important stride toward the discovery of candidate genes governing kiwifruit quality attributes and nutritional contents. Similarly, another GWAS focusing on commercially valuable fruit shape traits has successfully identified 115 significant SNPs and 349 candidate genes in a wild population of *A. eriantha*, providing molecular resources for marker-assisted breeding of kiwifruit with desirable fruit characteristics [135].

### Whole-genome comparative analysis

Since the first draft genome assembly (HongyangV1) was published in 2013 [12], large-scale comparative analysis has emerged as a pivotal tool for deciphering the evolutionary trajectory, speciation mechanism, and phenotypic diversification of kiwifruit. In the absence of genome sequences from congeneric species, initial comparative analyses were conducted across kiwifruit, Arabidopsis, rice, grape, and tomato, revealing an ancient hexaploidization event and two more recent duplication events that may occur in the evolutionary history of *A. chinensis* [12]. With the release of *A. eriantha* genome (WhiteV1) in 2019, systematic comparisons of gene family evolution between two closely related kiwifruit species became feasible, supporting their speciation timeframe at ~3.3 million years ago [17]. Meanwhile, inter-species comparisons can identify lineage-specific sequence variations, such as SNPs and structural variants (SVs) unique to particular taxa. This discovery provides valuable genetic resources for kiwifruit breeding and improvement, as *A. eriantha* exhibits strong resistance to *Psa*, a major threat in agricultural production [57].

More recently, breakthroughs in haplotype-resolved assembly can facilitate high-resolution comparative analyses of distinct haplotypes within the same genome. Notably, the haplotype-resolved T2T genome assembly (HongyangV4) of *A. chinensis* has revealed extensive genetic variations between haplotypes, including 3 950 488 SNPs, 808 012 InDels, 90 inversions, 1605 translocations, and 6120 duplications, which may contribute to the functional diversity of allelic genes [21]. Furthermore, phasing of HongyangV4 also permits genome-wide analyses of ASE and transcriptional allelic imbalance at the transcriptional level. In addition, accurate haplotype phasing is particularly critical for dioecious and allopolyploid genomes, as it enables allele-resolved characterization of SDRs and subgenome interactions. For example, the availability of phased diploid sex chromosomes directly exhibits a physically large hemizygous region that harbors the sex-determining genes and locates on the Y chromosome [23, 28]. Specifically, whole-genome comparative analyses across species uncover frequent inter-chromosomal translocations of the SDRs, indicating significant turnover of sex chromosomes during kiwifruit evolution.

### Graph-based pan-genome construction

Although multiple kiwifruit genome assemblies have been published to date, single linear references, even with phased haplotypes, inadequately represent the extensive genomic diversity within and between species, owing to numerous structural variants among cultivated varieties and wild accessions of the genus *Actinidia*. To address this limitation, pan-genomics has initiated a transformative research paradigm, which involves comparative genomic analysis across all related individuals [140, 141]. By integrating 14 chromosome-scale haplotype-resolved genome assemblies from 7 cultivated accessions of *A. chinensis*, the first kiwifruit pan-genome was constructed, providing a comprehensive framework to characterize both core genes/sequences (shared by all accessions) and dispensable genes/sequences (absent in at least one accession) [30]. As the selected accessions cover three fruit color categories (green, yellow, and red), it enables the identification of a 51-bp SV in the promoter region of *AcBCM* that regulates chlorophyll accumulation in the green-flesh fruits.

In contrast to the rich diversity of fruit colors observed in *A. chinensis*, *A. eriantha* consistently maintains a higher chlorophyll level during fruit ripening, resulting in a characteristic dark-green flesh phenotype. Leveraging the super pan-genome deposited in the KPGD, a novel SV overlapping the coding region of *PPR*, whose homologous gene in rice encodes a pentatricopeptide repeat-containing protein that functions in chloroplast development [13]. Beyond the well-studied flesh color, at least three published kiwifruit pan-genomes to date have systematically characterized other important traits by utilizing distinct genomes from the genus *Actinidia*, including fruit size, vitamin C content, and disease resistance [32, 33, 142]. Furthermore, by applying synteny-based comparative analysis to the super pan-genome of *A. chinensis* and *A. eriantha*, researchers have identified the conserved male-specific gene *YFT*, which fine-tunes flowering time and underlies the observed sexual dimorphism in kiwifruit [143]. Collectively, these studies have elucidated the genetic basis of key agronomic traits and enabled more precise marker-assisted selection strategies in kiwifruit breeding programs.

## Genome editing: from target discovery to trait engineering

The cascade of knowledge generated from multi-omics methodologies, genomic resources, and diversity studies has fundamentally shifted kiwifruit research from observation to intervention. Genome editing, particularly the CRISPR-Cas9 system, now serves as the pivotal tool to close this loop, enabling the direct functional validation of candidate genes and the precise engineering of agronomic traits [144]. To date, the most compelling applications of this transformative technology in kiwifruit are those precisely targeting its fundamental biological constraints: protracted juvenility and sexual dimorphism.

First, a landmark demonstration of this approach is the CRISPR-Cas9 knockout of *CEN-like* genes in kiwifruit [145]. Mutation of these flowering repressors resulted in dramatic phenotypic shifts, including very early reproductive maturation, increased determinacy, and a continuous flowering habit. Beyond confirming the conserved function of *CEN* genes, this study’s most impactful innovation is the material itself. The resulting compact, rapid-cycling plants constitute a novel model system that circumvents the traditional bottleneck of long juvenility, thereby enabling accelerated characterization of gene function, as exemplified by studies on the *Friendly Boy* (*FrBy*) gene governing androecia development [146] and the *Shy Girl* (*SyGl*) gene involved in suppression of feminization [147]. In contrast to the early-flowering phenotype achieved by editing *CEN* genes, mutagenesis of *BFT* genes results in enhanced, continuous vegetative growth without accelerating flowering. This contrast highlights the precision of genome editing in selectively manipulating distinct developmental pathways to meet different breeding objectives [148].

Furthermore, CRISPR-Cas9 has also been deployed to reprogram reproductive architecture. One study established a stable knockout by targeting two distinct sites of the *SyGl* gene to produce stable hermaphroditism within a male kiwifruit (*A. chinensis*) genotype [149]. Although confirmation of the resulting floral phenotype is pending due to the long reproductive cycle, this work represents a significant step forward. In itself, it demonstrates the critical need and substantial potential of using accelerated flowering genotypes to validate such traits efficiently. Separately, the development of early-flowering kiwifruit materials by other research groups stands as a direct response to this overarching challenge [145]. Utilizing such existing rapid-cycle models could potentially streamline the validation process for edited lines, as demonstrated in this case. Therefore, the systematic application of early-flowering materials in functional studies represents the logical next step to decipher the genetic networks controlling key reproductive and agronomic traits.

In parallel to the creation of novel germplasm and the characterization of gene function, another line of innovation focuses on advancing the methodological toolkit itself. A prime example is the establishment of a rapid marker-free transformation and highly efficient CRISPR-Cas9 editing system for kiwifruit [150]. To overcome the time constraints of genetic manipulation, this system leverages *Agrobacterium rhizogenes*–mediated hairy root transformation, coupled with a key technical step of root-tip removal to boost regeneration. Practical application confirmed its high efficiency, with editing rates of 50% to 55% and successful stable transformation across species. Moreover, the successful dissection of *CBL3* gene function in calcium oxalate formation showcases the direct impact of this methodological advance on accelerating functional genomics. By drastically improving efficiency, this system now constitutes a critical enabling platform for accelerating the exploration of gene functions linked to key horticultural traits [151].

Despite its promise, the deployment of genome editing in kiwifruit still faces some biological and technical hurdles. Foremost among these is the genotype-dependent transformation efficiency, which severely restricts the application of editing tools in commercial cultivars. Furthermore, the polyploid nature of these cultivars presents a significant barrier to complete gene knockout, necessitating advanced multiplex editing strategies for clear functional analysis. Looking ahead, emerging technologies, such as base editing and prime editing, offer the potential for precise allele creation without double-strand breaks [152]. This capability may prove especially valuable for introducing subtle, beneficial variations or correcting deleterious alleles in elite cultivars. A major step forward will be the integration of these next-generation editors with improved delivery methods (e.g. ribonucleoprotein complexes) to unlock the full potential of precision breeding.

Excitingly, genome editing is redefining the future of kiwifruit improvement. It transforms the vast datasets from genomics and phenomics into actionable genetic targets, enabling a shift from selective breeding based on chance recombination to rational design based on known function. As the regulatory landscape for edited crops clarifies, this technology is poised to advance beyond the proof-of-concept stage. Consequently, it is set to become a central component of kiwifruit breeding programs, thereby accelerating the development of elite cultivars with enhanced resilience, quality, and yield.

## Future directions: balancing challenges and opportunities

### Evolutionary forces driving sex chromosome turnover

Kiwifruit (*Actinidia* spp.) exhibits an XY sex-determination system, which has been consistently demonstrated through cytogenetic analyses in early studies and subsequently validated by different molecular marker systems [127, 138, 153, 154]. Unlike many animal Y chromosomes, the kiwifruit Y chromosome retains homologous recombination throughout most of its length, except for a small unpairing region that was further confirmed as the SDR [20]. However, due to the higher proportion of repetitive sequences in this region, complete sequence assembly and accurate gene prediction has been technically difficult for decades [155]. Recently, two Y-linked sex-determining genes, *Shy Girl* (*SyGl*) and *Friendly Boy* (*FrBy*), were successfully identified from the SDR through BSA-based genetic mapping coupled with transcriptome sequencing [146, 156]. Although these two genes are highly conserved across kiwifruit species, the SDRs where they are located exhibit significant variation in both sequence length and nucleotide composition [28]. Whole-genome sequencing and assembly demonstrated that LTR retrotransposon insertions contribute not only to the local SDR expansion, but also to the global polymorphism emergence after species divergence [23, 28].

**Figure 3:**
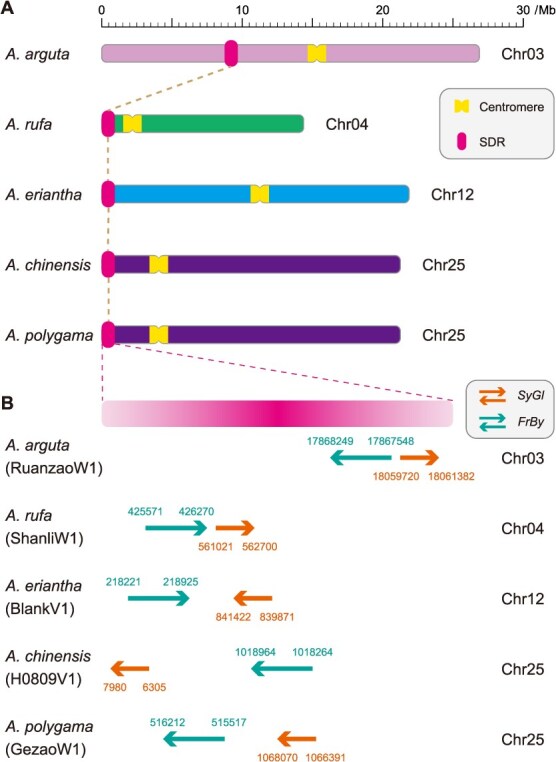
Location of the SDRs and sex-determining genes (*SyGl* and *FrBy*) on the Y chromosome in each species. (A) Chromosome structure is based on the HongyangV4 reference genome assembly, showing chromosome length, orientation, and centromere position. (B) Detailed view of each SDR, depicting the relative positions and transcriptional directions of *SyGl* and *FrBy*. Arrows indicate the transcriptional direction, with their respective start and end points numbered.

Notably, comparative genomic analyses have uncovered the frequent turnover events of sex chromosomes in kiwifruit (Fig. 3A). Meanwhile, common inversions and/or insertions of the *SyGl* and *FrBy* genes within the SDRs lead to no consistent pattern in their genomic locations across the five sequenced male species, even those sharing the homologous sex chromosome (Fig. 3B). Despite these phenomena are increasingly documented across studies, our understanding of the evolutionary force driving the recurrent sex chromosome turnovers and sex-determining gene relocations is limited. Furthermore, it is still unknown whether the inter- and intra-chromosomal translocations contribute to phenotypic diversification and/or speciation process. To address how, when, and why sex chromosome transitions occur, we require far more information on the evolutionary history of sex chromosomes and autosomes in understudied kiwifruit clades.

### Phylogenetic reconstruction based on large-scale genomic data

**Figure 4:**
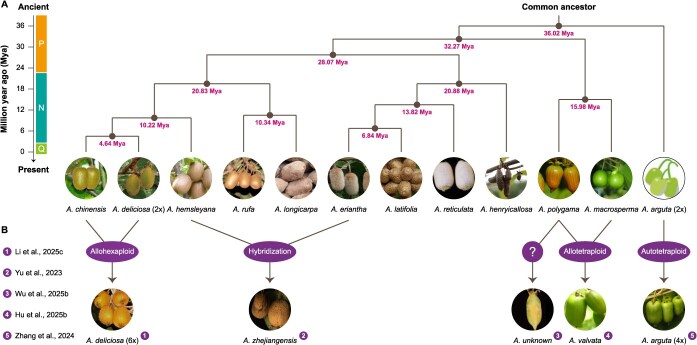
*De novo* reconstructed evolutionary model of all studied kiwifruit species. (A) The estimated divergence times shown at each node are inferred based on single-copy orthologous groups, using a Bayesian approach implemented in MCMCTree of PAML [160]. The letters P, N, and Q adjacent to the axis of time denote the Paleogene Period (66–23 Mya), Neogene Period (23–2.58 Mya), and Quaternary Period (2.58 Mya to present), respectively. (B) The depictions of duplication and hybridization events are based on a survey of the published literatures. Notably, *A. zhejiangensis* is identified as a transient dead-end hybridization.

Phylogenetic inference, the study of evolutionary relationships among organisms, plays a crucial role in tracing the origins of homologous genes, related species, and even entire ecosystems. But the construction of species tree could be confounded by reticulate evolutionary processes, particularly through hybridization and introgression events that violate the bifurcating tree model [157]. As interspecific hybridization occurs extensively in dioecious kiwifruit (*Actinidia* spp.), the resulting lineages in extant species often possess chimeric genomes with heterogeneous phylogenetic signatures. For example, by applying whole-genome sequencing to 25 kiwifruit accessions, researchers successfully reconstructed hybridization-mediated reticulate speciation patterns, enhancing our understanding of the ‘Web of Life’ metaphor [158]. Thus, accurate species tree construction is notoriously challenging when analyses rely on limited gene sets [159]. Alternatively, the integration of large-scale genomic data has revolutionized phylogenetic reconstruction, allowing both high-resolution species tree estimation and reliable dating of major evolutionary events (Fig. 4A). Conceivably, future advancements in data availability of more high-quality genomes should enable tracing evolutionary patterns of speciation-associated chromosome reorganization and species-specific gene flow following natural hybridization among the genus *Actinidia*.

### Re-estimation of species number in the genus *Actinidia*

Currently, kiwifruit (*Actinidia* spp.) comprises 54 recognized species based primarily on traditional morphological characters [2]. However, this taxonomic classification may represent an overestimation of true phylogenetic diversity. As noted above, while interspecific hybridization is pervasive in *Actinidia*, it does not invariably lead to successful speciation (Fig. 4B). If hybrids are non-viable or sterile, the resulting lineages typically represent evolutionary dead-ends [161]. Due to morphological distinctness, they are often misinterpreted as independent evolutionary lineages rather than ephemeral products of crossing, which can obscure true species boundaries in phylogenomic studies, such as the analysis conducted on the ‘Russell’ genome [20]. Another one representative case in kiwifruit involves the elucidation of phylogenetic placement and evolutionary origin for *A. zhejiangensis*, which was initially classified as a new species but now redefined as a homoploid sterile hybrid lineage based on whole-genome analyses [26, 162]. Furthermore, a super pan-genome constructed from all publicly available genomes has independently confirmed this dead-end hybridization event of *A. zhejiangensis*, revealing that its genome comprises one haplotype derived from *A. eriantha* and the other from *A. hemsleyana* [13]. Notably, the availability of large-scale genomic data has not only significantly improved the resolution and accuracy of phylogenetic constructions but also enabled the comprehensive revision of existing species trees, leading to paradigm shifts in our understanding of evolutionary relationships. Considering that the genomic resources for many kiwifruit species remain unavailable, we reasonably postulate that dead-end hybridization events may be more prevalent than currently documented, potentially leading to inflated species counts in the genus *Actinidia*.

### Molecular mechanism underlying the formation of polyploidy

In natural systems, kiwifruit (*Actinidia* spp.) exhibits multiple ploidy levels spanning from diploid (2*n* = 2*x* = 58) to decaploids (2*n* = 10*x* = 290), with a conserved base chromosome number of *x* = 29 [4, 163]. This may suggest that polyploidy serves as a significant evolutionary force driving both speciation and subsequent diversification. However, research on polyploidization has progressed slowly due to the inherent complexity of genome assembly, especially subgenome discrimination challenges posed by the high chromosome number. Recent breakthroughs in long-read sequencing (e.g. PacBio HiFi and Oxford Nanopore) and chromatin conformation capture (Hi-C) have facilitated chromosome-level and haplotype-resolved assembly of polyploid genomes, establishing a critical foundation for elucidating the polyploidization processes in kiwifruit [27, 29, 31, 35, 36]. Although these resources now enable systematic discrimination between autopolyploidy and allopolyploidy, the classification of hexaploid *A. deliciosa* remains debated [27, 36]. In the future, additional high-quality genome assemblies will enable subgenome ancestry tracing and help resolve whether polyploidization events resulted from auto- or allopolyploidy. Resolving these origins is a critical step toward elucidating the broader molecular and evolutionary mechanisms of polyploid formation in kiwifruit, particularly the factors that trigger genome duplication and the subsequent genomic changes. This, in turn, will allow for the systematic tracking of gene retention, loss, and functional diversification between subgenomes. Ultimately, such efforts are expected to uncover the genetic basis of how polyploidy has contributed to key agronomic traits, such as environmental adaptability and fruit quality, providing a deeper understanding of evolution-driven innovation in the genus *Actinidia*.

### The applications of integrative multi-omics technologies

**Figure 5:**
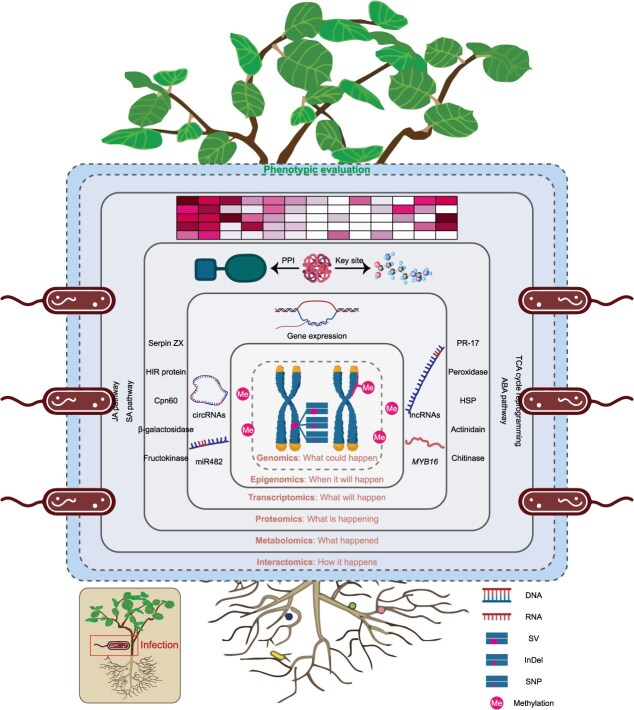
A multi-omics systems view of kiwifruit response to *Psa* infection. The diagram depicts the cascade of molecular events from the genome to the phenome upon *Psa* challenge. From the innermost to the outermost, concentric circles represent distinct yet interconnected omics layers: genomics (blueprint), epigenomics (switch), transcriptomics (message), proteomics (machinery), metabolomics (record), and interactomics (battleground). Bacterial silhouettes on the periphery illustrate the invasion of *Psa*, which perturbs these layers and triggers host defenses, leading to the resulting phenotype (resistance or susceptibility). The inset in the lower-left corner provides the biological context, showing the localized infection site on a kiwifruit plant. The illustration of the kiwifruit plant was redrawn based on the schematic presented by Zheng *et al*. [165].

Integrative multi-omics technologies represent a paradigm shift in systems biology, offering a holistic framework to decipher complex biological processes. In kiwifruit, while pioneering combined analyses, such as those linking transcriptomics with proteomics or metabolomics, have begun to emerge [62, 63, 67, 164], their scale and interpretive depth remain limited. Research on kiwifruit bacterial canker represents one of the most data-rich areas. Given its economic importance, *Psa* has been extensively investigated across multiple omics layers (Fig. 5). Specifically, genomics, transcriptomics, proteomics, and metabolomics have advanced our understanding by identifying putative resistance loci, profiling defense responses, and cataloging changes in protein and metabolite abundances. However, the insights from these individual or pairwise omics studies remain isolated and thus cannot construct a mechanistic model linking genetic variation to the specific molecular recognition events underlying immunity. For instance, while a resistance QTL may be identified, the epigenetic modification controlling its expression, the causal proteins it encodes, the metabolites it ultimately influences, and how these collectively determine the final disease phenotype are rarely connected systematically. This fragmentation hinders the identification of the key regulatory nodes and dynamics within the resistance mechanism. Furthermore, host-pathogen interactomics, which aims to map the comprehensive network of molecular interactions during infection, has not yet been systematically applied to the kiwifruit-*Psa* system. Therefore, establishing causality, such as linking a metabolite shift to a specific enzyme encoded by a differentially expressed gene within a QTL, requires tightly coupled multi-omics data from identical samples under controlled conditions.

Moving forward, leveraging integrated multi-omics approaches will be essential to unlock the molecular networks governing fruit development, quality traits, shelf life, as well as responses to both biotic and abiotic stresses at a systems level. This integrated approach, as evidenced by its mature application in species such as apple, grape, and potato [166–169], will undoubtedly open new avenues for exploration in kiwifruit research. On a practical level, translating these insights into breeding is central to enhancing kiwifruit productivity and resilience. This synergy will not only drive the development of better computational tools and collaborations but also propel multi-omics into broader agricultural applications.

### Genomic selection for accelerating breeding programs

Kiwifruit breeding is a slow process and usually faces biological and logistical challenges, such as long juvenile periods, polyploidy, and complex inter- and intra-specific hybridization. Marker-assisted selection (MAS) can significantly shorten breeding cycles, boost genetic progresses and accelerate crop improvement by predicting breeding values from genome markers rather than waiting for full phenotypes. In kiwifruit, early sex determination stands as the most successful and transformative application of MAS, reducing the duration for sex identification from multiple years to mere months [127, 170, 171]. Meanwhile, the application of best linear unbiased prediction allows direct estimation of sex-related traits to speed up the breeding process [172]. However, the traditional MAS is not well suited for complex traits that are controlled by numerous small-effect loci, such as fruit quality, disease resistance, stress tolerance, and total yield [173].

**Figure 6:**
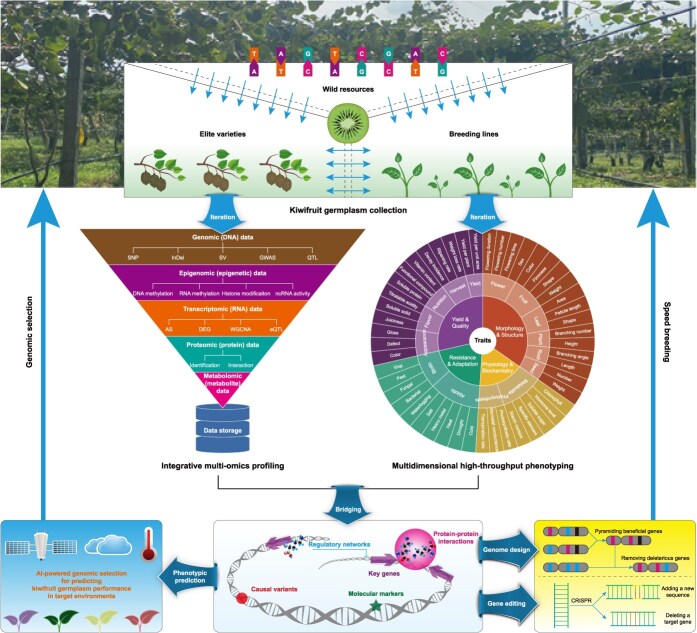
A vision for the integrated intelligent breeding workflow in kiwifruit. This workflow outlines a forward-looking paradigm for kiwifruit improvement, with speed breeding and genomic selection as the core engines. It begins with the high-throughput collection of genomic and phenomic data from diverse germplasm, including wild resources, elite varieties, and breeding lines. Candidate genetic elements, such as key genes, causal variants, molecular markers, and regulatory networks, are then identified for genome design and gene editing. The entire process, combined with target environmental information, is leveraged by AI-driven models to predict breeding values, which can accelerate the development of new varieties with enhanced crop performance. These improved varieties, in turn, feed back into the cycle as new breeding parents, creating a self-reinforcing ‘breeding flywheel’ that enables continuous learning and optimization of the prediction models. Together, this closed-loop, data-driven system aims to drastically increase the speed and precision of breeding new, superior kiwifruit varieties.

Genomic selection (GS), an advanced form of MAS simultaneously employing thousands of genome-wide markers to calculate breeding values, can demonstrate optimal efficacy for polygenic trait improvements. In kiwifruit, GS has been primarily explored in diploid *A. chinensis* for complex traits such as yield, flesh red color intensity, and fruit firmness [174]. More recently, it has also been applied to estimate heritabilities and predict traits associated with flower load in autotetraploid *A. arguta* [175], and to correct pedigree errors and evaluate prediction accuracies for many vine and fruit traits, including fruit load, fruit weight, dry matter percentage, and ripe soluble solid content, in both intra-specific (*A. arguta* × *A. arguta*) and inter-specific (*A. arguta* × *A. melanandra*) kiwifruit breeding populations [176, 177]. These efforts have initiated GS in kiwifruit research, yet they remain nascent when compared to the well-established, large-scale research systems in major staple crops [178–180]. Currently, the integration of multi-omics profiling and high-throughput phenotyping offers unprecedented opportunities for dissecting and leveraging the genetic basis of important agronomic traits, ultimately empowering precision breeding of improved kiwifruit cultivars. Empowered by artificial intelligence (AI) and gene editing (GE), kiwifruit breeding can now also enter the era of intelligent breeding (Breeding 5.0) (Fig. 6).

As a perennial vine crop with long generation times and complex ploidy variations, kiwifruit should stand to benefit significantly from Breeding 5.0 to accelerate genetic gain. Furthermore, a substantial number of understudied species in the genus *Actinidia* critically serve as indispensable germplasms for genomic-assisted breeding and elite cultivar development. Despite current limitations in prediction reliability and large-scale implementation, the rapid maturation of core technological drivers, including genomic and phenomic characterization, genome design, synthetic biology, precision editing, and AI-driven analytics, alongside the development of collaborative frameworks, will undoubtedly establish GS as a fundamental pillar of modern kiwifruit breeding programs in the foreseeable future.

## Supplementary Material

Web_Material_uhag024
